# The psychological impact of the COVID-19 pandemic on children/adolescents with ASD and their family environment: a systematic review

**DOI:** 10.1007/s00787-023-02151-6

**Published:** 2023-02-09

**Authors:** Andreea Cristiana Milea-Milea, Dolores Fernández-Pérez, Abel Toledano-González

**Affiliations:** 1 General University Hospital of Castellón-Fisabio, Castellón de la Plana, Spain; 2 Department of Psychology, Faculty of Medicine, University of Castilla-La Mancha, Albacete, Spain; 3 Department of Psychology, Faculty of Health Sciences, University of Castilla-La Mancha, Talavera de la Reina, Spain; 4 Neurological Disabilities Research Institute, Albacete, Spain

**Keywords:** Autism spectrum disorder (ASD), COVID-19, Family environment, Pandemic, Psychological impact

## Abstract

The main objective of the review is to analyze the impact of the pandemic in children/teenagers with Autism spectrum disorder (ASD) and in their family environment. An electronic search was carried out in different databases (PsycInfo, Web of Science and Scopus) in order to find publications associated with the aim of this project. The search terms used were derived from the combination of the following search string: “((Pandemic OR Epidemic OR Outbreak OR COVID-19 OR Coronavirus) AND (Children OR Adolescents OR Youth OR Child OR Teenager) AND (Autism OR ASD OR Autism Spectrum Disorder))’’. In total, 21 articles were included in this review. The findings of the different investigations included in this review show that the pandemic has produced a negative psychological impact in children/teenagers with ASD. Therefore, long-term follow-up studies should be carried out with the objective of creating effective interventions to treat this problematical.

**Supplementary Information:**

The online version contains supplementary material available at 10.1007/s00787-023-02151-6.

## Introduction

Autism spectrum disorder (ASD) is a neurological and developmental disorder characterized by difficulties in social interaction and communication, as well as restricted interests and repetitive behaviors with different degrees. This pathology begins before the age of 3 years [19]. With the publication of the Diagnostic and Statistical Manual (DSM-V) in 2013, aspects related to the previous classifications proposed by the DSM-IV-TR (fourth revised edition) have been modified. Thus, in the DSM-V, the various autism spectrum disorders that were included in the DSM-IV-TR (autistic disorder, Asperger syndrome, pervasive developmental disorder not otherwise specified, Rett syndrome and childhood disintegrative disorder) have been unified and consolidated into a single umbrella diagnosis of autism spectrum disorder (ASD) [4].

Likewise, authors such as Vázquez-Villagrán et al. [48] have determined this pathology as a neurological variation disorder of multifactorial origin that affects the development of socioemotional and behavioral skills, with the presence of repetitive and restricted behaviors and interests. The course of this neurological and developmental disorder is chronic at the linguistic and intellectual functional level, depending on the case, the developmental stage and the level of affectation [53].

The diagnosis of this disorder not only limits the quality of life of children, but also indirectly impacts their environment. Parents of children with ASD present higher levels of stress and also an increase in marital conflicts. In addition, these parents often report depressive and anxious symptoms due to the high caregiving demands required by children with ASD [10, 22, 31]. More specifically, some studies have found that parents of children with ASD experience higher levels of stress compared to parents of children with other types of developmental disorders (intellectual disability, specific learning disorder, attention deficit hyperactivity disorder, etc.) [15, 21].

Based on the above, caring for a child with ASD is, in some cases, stressful for their caregivers. Moreover, this effect has been increased due to COVID-19, a virus that the World Health Organization qualified as a pandemic in March 2020 [51]. The arrival of this pandemic has led governments to take containment measures to control the virus such as school closures, social distancing, and home quarantine, creating a high degree of disruption in the lives of children with ASD and their families [36].

In addition, several studies [13, 16, 44] state that most families with children with neurodevelopmental disorders, had problems managing their children's daily behaviors and activities during the pandemic compared to the time before this. In addition, depressed mood, distress, repetitive behaviors, anxiety, and frustration during confinement have been found to be increased in children with autism, making it difficult for their parents to cope with such behaviors [2, 6, 27, 29].

Similarly, in a study done by Dhiman et al. [17] in which is analysed the mental health and the stress of parents with children with special educational needs (SEN), the results show that the parents have been experienced higher levels of depressive, anxious and stress symptoms during the pandemic. The increase of this symptomatology is having been associated with a lack of the follow-up diagnosis and treatment of their children by social-health centers or educational centers. Continuing along these same lines, families with children diagnosticated with ASD have experienced higher level of stress, even when the therapies have been adapted online due to the inadequacy of the services.

To conclude, it can be observed that the pandemic has meant a radical change in people's lives, especially in children with neurodevelopmental disorders and their families. For this reason, the main objective of this review is to analyze in detail the consequences that the pandemic produced by COVID-19 has had on children/adolescent with ASD and their family environment.

Therefore, this review is also necessary to explore the anxiety, depression and the parental stress during the pandemic. Moreover, this review will provide relevant information about the behavioral changes that have occurred in this population with ASD during the pandemic. Finally, through this study, it will also be possible to observe if the pandemic has generated positive effects in children or adolescents with ASD.

## Materials and methods

The present systematic review was conducted in accordance with the Preferred Reporting Items for Systematic Reviews and Meta-Analysis (PRISMA) guidelines [38]. The protocol of this systematic review was registered with PROSPERO (International Prospective Register of Systematic Reviews) before starting this review (registration number CRD42022317548).

### Search strategy

To carry out the applicability of this systematic review, a search strategy was carried out on the Web of Science (WOS), PsycInfo (American Psychological Association) and Scopus databases until January 2022. The inclusion and exclusion criteria are shown in Table 1. The search string was applied to the fields Title + Summary + Keywords using the combination of the following keywords: “((Pandemic OR Epidemic OR Outbreak OR COVID-19 OR Coronavirus) AND (Children OR Adolescents OR Youth OR Child OR Teenager) AND (Autism OR ASD OR Autism Spectrum Disorder))’’.

**Table 1:** Inclusion and exclusion criteria for studies

Inclusion criteria	Exclusion criteria
Articles published between 2020 and 2022	Wrong study population
Articles in English and Spanish	Articles from other disciplines that do not correspond to the field of psychology
Articles whose study population was children and adolescents with Autism Spectrum Disorder (from 3 to 18 years of age) or family members/caregivers of this population	
Articles that analyze the impact of the pandemic in the context of the family	

### Selection of studies

To select the articles, a web application called Covidence (a systematic review program that aims to improve evidence synthesis, www.covidence.org) was used. Potentially eligible articles had to present original research on the topic chosen for the review. Only articles published in scientific journals were selected. Gray literature was excluded.

Studies based on the approach to children or adolescents with ASD and the effects of the pandemic on their autonomy and personal independence were selected. Articles were excluded for the following reasons: (1) articles that did not analyze psychological, behavioral and affective-emotional aspects of the pandemic in children and adolescents with ASD; (2) articles that did not include cross-sectional/longitudinal studies; (3) articles where the population was not children and adolescents diagnosed with ASD or their relatives; (4) articles that did not consider the impact of the pandemic in the family environment.

The minimum sample size of the studies was not restricted. Two independent reviewers (ACMM and ATG) selected studies and extracted relevant data. Discrepancies were resolved by a third independent review (DFP).

### Data extraction and analysis

Two investigators (ACMM and ATG) conducted the literature searches and reviewed the relevance of titles and abstracts following inclusion and exclusion criteria, in addition to data extraction. Clinical efficacy was defined in terms of whether study results showed that a test or treatment resulted in symptom improvement. A data extraction form was developed to extract the most important data, including the following: article title, year, authors, study type, sample size, follow-up, participant characteristics, intervention description (if any), main results, and conclusions.

In the articles reviewed, objective measures of effect were used to assess the impact of pandemic/confinement on the ASD population. The main variable, psychological variables, and behavior were analyzed from different perspectives with the aim of verifying the processes occurring with respect to family dynamics. This can be seen in more detail in Table 2.

**Table 2:** Results of the main studies

Authors (year)	Quality Assessment	Total (N)	Study Type	Aim of the study	Instruments	Methodology/Data Collection	Results	Conclusions
Alhuzimi [2]	7/8	N = 150 parents of ASD children	Cross-sectional study	Investigating the stress and emotional well-being of parents of children with ASD during the COVID-19 pandemic	Questionnaire composed of 6 parts: Demographic data Family status Support received during the pandemic Severity of ASD Behaviours in comparison to the pre-COVID-19 status PSI-SF (Parental Stress) GHQ-12 (Emotional well-being)	Participants were given a brief summary of the objectives of the study and were informed that their responses would be used exclusively for the research purposes. Data collection was performed between 15nd June 2020 to 30th June 2020 using an online questionnaire that was sent to all participants by email	Severity of ASD symptoms was significantly related to parental stress (*F*/*t* = 5.562; *p* = 0.005), and also to parental-child dysfunctional interaction (*F*/*t* = 6.590; *p* = 0.002) Parental stress was significantly depending on the frequency and usefulness of support received during the pandemic (*F*_(3, 145)_ = 2.729, Wilk’s Λ = 0.947, partial *η*^2^ = 0.053) With respect to emotional well-being, the supports received had a positive impact on it. There was also a positive correlation between stress and parental emotional well-being	The stress and emotional well-being of parents with ASD children has deteriorated significantly during the COVID-19 pandemic
Althiabi [3]	7/8	*N* = 211 parents of children with ASD	Cross-sectional study	Assess the attitude, anxiety and care provided by mental health services in parents with ASD children during the COVID-19 pandemic	Questionnaire composed of 5 sections: Sociodemographic characteristic Parents’ attitude HADS (State of anxiety) GHQ-12 (Mental health status) Perceived mental health care need	Participants were informed of the purpose of the study, as well as the right to withdraw from the study at any time. Data collection was carried out through a questionnaire distributed online to participants during the months of June and July 2020	The general attitude towards COVID-19 is not different between fathers and mothers of children with ASD (*t* = − 1.043; *p* = 0.298) Anxiety: parental anxiety was found to be significantly increased compared to pre-COVID anxiety level (*t* = – 7.551; *p* = 0.000) Significant moderate positive relationship between attitude and state of anxiety during COVID-19 (*r* = 0.302; *p* < 0.010) Significant negative correlation between anxiety and mental health status (*r* = – 0.430; *p* < 0.010) Significant negative correlation between the perception of attention from mental health services and anxiety (*r* = – 0.317; *p* < 0.010)	The COVID-19 has impacted on the attitude, anxiety and mental state of parents with ASD children as well as their perception of the necessity of mental health services
Amorim et al. [5]	7/8	*N* = 99 (*n* = 43 children with ASD, *M* = 9.86, SD = 3.08 and *n* = 56 children without neurodevelopment disease (group control), *M* = 11.43, SD = 3.03)	Observational, cross-sectional and analytical study	Analyze how children with ASD and their parents experienced the social isolation in their homes during school closedown in the COVID-19 outbreak	Questionnaire developed by the authors of the article with the aim of exploring the demographic and clinical characteristics of the sample, as well as the impact of social isolation in the home during the COVID-19 outbreak	The information was obtained by telephone, as well as through an online survey during April 2020. In addition, all participants in the study accepted the informed consent	Parents of children with ASD predominantly reported changes in behavior of their child, while parents of children from control group mostly found no changes, and differences between these groups were statistically different (*p* < 0.050) Parents from both groups reported more negative than positive impact of quarantine in learning, but there weren’t statistically significant differences between the groups (*p* = 0.572) In terms of emotion management, the impact difference of quarantine was significant between the two groups (*p* = 0.020). The majority of parents with ASD children reported a negative impact on emotion management. Higher levels of anxiety in children with ASD, as well as in their parents compared to the control group. The differences between both groups were statistically significant (*p* < 0.050)	After the results obtained, health professionals should pay attention not only to the groups at risk, but also to their family members
Bentenuto et al. [7]	7/8	*N* = 164 parents (82 parents of children with neurodevelopmental disabilities (*M* = 7.63, *S* = 3.77) and 82 parents of typically developing children (*M* = 7.67, *S* = 3.86))	Cross-sectional study	Assess parental stress and children's adaptation during confinement	Sociodemographic information PSS CRS SDQ_ext Question developed by the authors to evaluate the follow-up medical care compared to the period prior to confinement Question elaborated by the authors: *''Please describe the most significant aspect of your experience as a parent during COVID-19''*	The population of interest was recruited through snowball sampling. Data was collected through an online survey with a duration of 15 to 20 min	Higher parental stress in families of children with neurodevelopmental disorders (*F*_(1.162)_ = 4.46; *p* = 0.036) An increase in externalizing behaviors in children with neurodevelopmental disorders compared to the group of children with typical neurodevelopmental disorders (*F*_(1.162)_ = 10.53; *p* = 0.001) Significant correlation between children's externalizing behaviors and parental stress Negative correlation established between externalizing behaviors and therapy received	The results obtained suggest paying more attention to the psychological well-being of family members with children diagnosed with neurodevelopmental disorders in confinement
Berard et al. [8]	7/8	*N* = 239 subjects with ASD, *M* = 9.1, SD = 4.0	Cross-sectional descriptive and analytical study	Examine the impact of the measures imposed by the pandemic on the behaviors, communication, sleep and nutritional status of children and adolescents with ASD	Questionnaire designed by the authors of the article, composed of four sections: Family environment Parent professional status Level of information about COVID-19 Child status Scale developed by the authors of the article for parents to describe their child's behavior VABS-II ADOS-2 BL-R BECS PEP-3 WPPSI-IV WISC-V WAIS-IV K-ABC	Data collection through an online questionnaire sent by mail to parents of children with ASD. The information was collected between April 27 and May 13, 2021	Half of the parents reported no change in their children's sleep, communication skills and stereotyped behaviors. In addition, 28.8% of parents perceived an improvement in their child's communication skills. On other hand, there were no changes in the children's nutrition during confinement. However, 64.4% of parents reported that their children's defiant behaviors had increased during confinement	Although confinement had negative effects on children and adolescents with ASD, they showed an improvement in communication
Chan and Fung [11]	7/8	*N* = 129 (51 parents of children with neurodevelopmental disabilities (*n* = 15 ASD) and 78 parents of typically developing children), *M* = 41.2, SD = 6.56	Cross-sectional study	Investigate and compare the prevalence of COVID-19 related stress and mental health problems between parents of children with developmental disorders and those of children with typical development	Questionnaire developed by the authors to measure stress related to COVID-19 PHQ-9 (Depressive symptoms) GAD-7 (Anxiety symptoms)	Data collection through an online questionnaire provided by the research website. Confidentiality and anonymity of all participants was guaranteed	Parents with children with developmental disorders showed significantly higher levels of stress (*t* = 4.54; *p* < 0.001), depressive symptoms (*t* = 3.84;* p* < 0.001) and anxiety symptoms (*t* = 3.62; *p* < 0.001) compared to parents of children with typical development	The study's findings demonstrate that parents of children with developmental disorders report higher levels of stress, which, in time, leads to depressive and anxious symptomatology
Chen et al. [12]	7/8	*N* = 1450 parents of children with special needs (*n* = 454 parents of children with ASD), *M* = 40.76, SD = 5.84	Cross-sectional study	Analyze how the pandemic has affected the mental health of parents of special needs children	Demographic information Questionnaire developed by the authors to explore behavioral problems of children during COVID-19 Questionnaire done by the authors in order to analyze the psychological demands of the parents during COVID-19 GHQ-12 PSS PSI-SF-15 NEO-FFI	Data collection was conducted through an online, anonymous, voluntary questionnaire between February 18 and February 22, 2020. Participants were included in the study using a stratified random sampling method, recruited through special education schools	Parents of children with autism spectrum disorder were more likely to have mental health problems compared to parents of children with a visual or hearing impairment (*p* = 0.03) Also, the mental health of these parents was positively related with: Behavioral problems in children (*r* = 0.22; *p* < 0.001) Psychological demand of parents (*r* = 0.31; *p* < 0.001) Parenting distress (*r* = 0.38; *p* < 0.001) Parent–child dysfunctional interaction (*r* = 0.36; *p* < 0.001) And negatively related with: Family support (*r* = – 0.23; *p* < 0.001) Friend support (*r* = – 0.18; *p* < 0.001)	This study has shown that there are significant differences in the mental health of parents depending on the disorder presented by their children
Friesen et al. [18]	7/8	*N* = 616 parents of children with ASD	Cross-sectional study	Explore caregivers stress, anxiety and resilient coping during COVID-19	Demographic Information Questions elaborated by the authors of the article related to the difficulties experienced by the family members due to COVID-19 PSS-10 STAI-6 BRCS	Participants were recruited through social media and autism agencies. Study data collection began on June 9th, 2020 and ended on July 30th, 2020. Respondents were asked to complete a short online survey delivered through the [affiliation’s] survey tool (Qualtrics)	The 85.2% of caregivers showed moderate stress The 67.7% of caregivers showed high anxiety The 48.9% of caregivers showed low resilience Caregiver gender (*p* = 0.001), and caregiver marital status (*p* = 0.010) emerged as demographic factors significantly correlated with perceived stress The presence of mental health problems in the children before the pandemic correlated positively with parental stress (*p* = 0.001) Economic problems (*p* < 0.001), as well as having more children diagnosed with ASD (*p* = 0.002) correlated significantly with parental stress	The results indicate the need for psychological interventions designed to ameliorate the mental health consequences of COVID-19
Guller et al. [20]	7/8	*N* = 299 children and adolescents with neurodevelopmental disorders (*n* = 131 children and adolescents with ASD) *M* = 10.32, SD = 4.57	Cross-sectional study	Investigate the emotional and behavioral responses of children with neurodevelopmental disorders and their parents during the COVID-19 pandemic and the associated factors	Questionnaire prepared by the authors of the article, which included the following sections: Sociodemographic information Parental attitude about the pandemic Behavioral, emotional, sleep, and appetite problems in children/adolescents HADS (Anxiety and Depression)	Data collection through an online questionnaire sent to parents by email. The data acquisition was carried out between April 18 and 30, 2020	Emotional problems of children/adolescents with ASD: Irritability (40.45%) Unhappiness (26.7%) Behavioral problems of children/adolescents with ASD: Stereotypies (50.4%) Hyperactivity (46.6%) Significant correlation between parents' emotional problems and children's/adolescents' emotional/behavioral problems. (*p* < 0.001)	This study concludes that it is important to conduct additional research that analyzes the effects of the pandemic on this population in order to develop health practices appropriate to the needs of each child
Hosokawa et al. [23]	7/8	*N* = 84 children with ASD (*M* = 11.6, SD = 3.1) and 361 children with typically developing (M = 11.2, SD = 3.4)	Cross-sectional study	Compare differences in the prevalence of stress, stressors factors and behavior between children with ASD and typically developing children	Questionnaire elaborated by the authors and composed of the following categories: Demographic information Questions related to the psychological impact of the pandemic	Data collection through Google Forms from April 30 to May 8, 2020. Only mothers were able to answer the questionnaire, as they were more sensitive to their child's behavioral changes	In terms of the stress variable, there were no significant differences between the two groups (*p* = 0.744). However, a higher percentage of children in the ASD group were reported to be frustrated by the changes in their routine Situational understanding was less in the ASD group vs. the control group Increased restricted and repetitive behaviors in the ASD group (*p* < 0.010)	The study findings revealed that children with ASD were affected differently by the pandemic compared to typically developing children. Further research into the long-term effects of the pandemic on this population should be conducted in the future
Kawaoka et al. [28]	7/8	N = 121 children with special needs (n = 88 children with ASD, M = 11.0)	Cross-sectional study	Examine the behavioral and emotional changes of children with special needs before and after school closure due to COVID-19	Questionnaire elaborated by the authors of the article (school attendance, place where he/she spent time during school closures…) CBCL	Data collection via a questionnaire sent to parents on May 15, 2020	Increased externalizing behavior and aggressiveness (*p* < 0.010), and also problems related to thinking (*p* < 0.050)	Due to the increase in externalizing and aggressive behavior, the authors suggest that it is necessary to provide appropriate support that takes into account the characteristics of each person in a pandemic situation
Levante et al. [32]	7/8	*N* = 120 children (*n* = 53 children with ASD, *M* = 6.94, SD = 1.6 and *n* = 67 children with typically neurodeveloping, *M* = 8.47, SD = 1.04)	Cross-sectional study	Explore the impact that parental distress has had on the adaptive behavior of their children	Questions created by the authors related to COVID-19 exposure DASS-21 Self-designed questions about children's emotional and behavioral responses Questions made by the authors to explore behavioral problems before and during lockdown	Data were collected during the first mandatory lockdown due to COVID-19 in 2020 through an online survey disseminated on social networks and also by the snowball sampling technique. All participants accepted the informed consent before completing the survey	Parental distress significantly and negatively correlated with children’s positive emotions (r = −0.391; *p* = 0.004) Children’s positive emotions were significantly and positively related to their adaptive behavior (*β* = 0.347; *p* = 0.046) Significant differences were found in the behavioral problems of ASD children before and during confinement (stereotypies and repetitive behaviors increased during confinement) Higher distress experienced by parents of children with ASD compared to parents of typically neurodeveloping children	It is concluded that the pandemic caused by COVID-19 has increased the distress of families of children with ASD, as well as their stereotyped and repetitive behaviors compared to the pre-covid era
Lim et al. [33]	7/8	*N* = 107 caregivers of children with special needs (M = 7.6, SD = 4.1)	Cross-sectional study	Analyze if the pandemic has produced depressive and anxious symptomatology in caregivers of children with special needs	Questionnaire made by the authors DASS-21 CD-RISC 25	Data collection through an online questionnaire. Participants were allowed to share the questionnaire link to other caregivers of children with special needs. Data collection took place from mid-May to the end of June 2020	About 79.4% of caregivers were concerned that their child would regress or stagnate developmentally The 68.2% showed higher levels of stress compared to the pre-pandemic era The resilience of these caregivers was also affected by the effects of the pandemic	Through this study it is observed that caregivers of children with special needs showed higher scores in anxiety, depression and stress compared to the general population
López-Serrano et al. [34]	8/8	*N* = 441 parents of children and adolescents with pre-existing psychopathology (*n* = 91 parents of children with ASD)	Cross-sectional study	Examine the evolution of various clinical symptoms during lockdown and the coping strategies used by a sample of children and adolescents with pre-existing psychopathology	The Scale of Confinement and Psychological Impact on Children and Adolescents created by four clinical psychologists, a psychiatrist and a mental health nurse	Data collection through an online questionnaire using the Lime Survey application (May 11–14, 2020). Data were encrypted and assigned anonymous numeric codes, in accordance with data protection law	Children/adolescents with ADHD, ASD and Anxiety Disorders showed an increased frequency of self-injury and regressive behaviors compared to those with Affective Disorders. In addition, ASD children/adolescents presented higher scores in obsessive–compulsive symptomatology and stereotyped movements compared to other diagnoses However, having relatives in frontline (*r* = 0.10; *p* = 0.050) and a greater loss of income (r = 0.16; *p* = 0.040) correlated with a higher level of caregivers’ total stress, which, in turn, correlated with a greater impact on the symptoms of children and adolescents (*r* = 0.41; *p* < 0.001)	Through this research, the groups that showed the greatest increase in their own psychopathological symptomatology were autism disorders and conduct disorders. Confinement as well as measures adopted by the government were especially prejudicial to patients diagnosed with ASD
Meral [37]	7/8	*N* = 32 mothers (*M* = 37.4, SD = 7.38) and pathers (*M* = 39.34, SD = 10.62) of children and adolescents with ASD and neurodevelopmental disorders	Cross-sectional study	Investigate the impact that the confinement caused by the pandemic has had on the family context of children with ASD and neurodevelopmental disorders	Socio-Demographic Form Semi-Structured Interview about the COVID-19 pandemic BFDS FQOL A single-item question about family quality of life	Data collection through interviews conducted via conferences with the children's families. Data collection began on April 13 and ended on May 9	A significant negative correlation between family distress and family quality of life (*r* = − 0.38*; *p* < 0.050) A significantly positive correlation between family quality of life and family happiness (*r* = – 0.54**, *p* < 0.010) There was no significant correlation between family distress and family happiness (r = – 0.17, *p* = 0.330)	The study showed that the pandemic has had negative as well as positive effects in the family context
Mumbardó-Adam et al. [40]	7/8	*N* = 47 parents (*M* = 41.3, SD = 6.2) of children with ASD (*M* = 7.3, SD = 3.4)	Cross-sectional study	Analyze the type of needs that children and adolescents with ASD have presented during confinement	Questionnaire elaborated by the authors of the article with four sections: Socio-demographic information Child’s behavior and emotional changes Family status Type of support received (school, medical centers…)	Families were contacted through different associations involved in the care of children with ASD. The link to the questionnaire and the informed consent form were sent by mail. Data collection was carried out during the month of April 2020	New behavioral patterns appear in children during confinement.: Higher levels of autonomy in taking care of themselves (14.9%) Better communication (19.2%) Increase of the participation in the family environment (27.7%)	The results of this research show that the children and their families coped better than expected with the confinement
							Emotional status: Happier and calmer children compared to before confinement (40.4%) Irritability (23.4%) Sadness (8.5%)	
Mutluer et al. [41]	7/8	*N* = 87 adolescents with ASD, *M* = 13.96, SD = 6.1	Cross-sectional study	Investigate how children with ASD responded to the COVID-19 pandemic	Sociodemographic form Pandemic-related questions ABC PSQI BAI	Data collection through an online questionnaire that also included informed consent	55% of the parents reported that their child became more aggressive 26% of the parents reported an increase in their child's tics, as well as the appearance of new tics 29% of the parents reported that their child's social and communication skills had been affected 44% and 33% of parents also commented that there were changes in sleep and appetite, respectively 25% of family members experienced severe anxiety symptoms during the pandemic	Through this study, it has been possible to conclude that, in people with ASD, the pandemic has had a negative impact, which has also led to an increase in the anxiety of family members
Pecor et al. [42]	7/8	*N* = 580 caregivers (*M* = 41.9) of children with neurodevelopmental disorders and children with typically neurodeveloping	Cross-sectional study	Assess the quality of life of caregivers of children with ADHD and/or ASD before and during the pandemic compared to caregivers of typically neurodeveloping children	PedsQL FIM version 2.0	Recruitment of participants by convenience sampling and data collection through an online questionnaire during the month of June and July 2020	Family caregivers of all children (Typical Development, ADHD, ASD AND ADHD + ASD) reported that their quality of life had decreased compared to the pre-covid era (*p* < 0.001). In addition, caregivers of children with ADHD and/or ASD showed significantly lower quality of life compared to caregivers of typically developing children	From the results obtained, it is concluded that the pandemic has had negative effects in the family context
Ren et al. [43]	6/8	*N* = 1049 mothers, *M* = 45.76, SD = 9.45 and 402 fathers, *M* = 44.83, SD = 9.10 of children with special needs	Cross-sectional study	Examine through a survey the level of anxiety, stress and perceived support of parents of children with special needs	Demographic information CMBP PMBP PSI-SF-15 MSPSS NEO-FFI S-AI	Participants were sampled through stratified random sampling and recruited through various special education schools. As for the data collection, it took place between February 18 and 22, 2020 through an online questionnaire	There was no significant difference between parental gender and anxiety (*p* > 0.050) Higher levels of education were associated with lower levels of anxiety. Unemployed parents showed more significant anxiety than those who kept their jobs Parents of children with ASD showed significant anxiety A higher lack of social support combined with other factors produced higher levels of parental stress (*p* < 0.001)	The results confirmed how stress, social support and other factors influenced parents' anxiety levels
Siracusano et al. [45]	8/8	*N* = 85 (*n* = 33 preschool children with ASD, *M* = 4 y *n* = 52 school children with ASD, *M* = 9)	Cross-sectional study	Examine changes in adaptive functioning, as well as to look at problem behaviors that have emerged after confinement by comparing data collected pre- and post-pandemic in preschool and school children with ASD	Leiter-R WPPSI-III o WISC-IV ABAS-II ADOS-2 RBS-R CBCL	Data collection via telephone after accepting informed consent	After confinement, a significant improvement was observed in the adaptive functioning of preschool children, with the exception of the social area (*t* = 1.62; *p* = 0.115). However, in school children, no significant results were found in pre- and post-pandemic adaptive behavior (*p* > 0.050) As for repetitive and disruptive behavior, there was no significant deterioration in either group (*p* > 0.050)	Despite the results obtained, it is not possible to analyze the effects in the long term, even so, is a study with high replicability
Wang et al. [49]	8/8	*N* = 6726 (*n* = 1764 parents of children with ASD and *n* = 4962 parents of children with neurotypically developing)	Cross-sectional study	Analyze the psychological distress, coping strategies, resilience, depressive and anxious symptomatology of parents with children with ASD during the pandemic	Sociodemographic information PSCQ CD-RISC SCSQ SAS SDS	Data collection through an online questionnaire sent by teachers to parents of children during March and April 2020	Parents of children with ASD obtained lower scores in resilience compared to parents of children with typical neurodevelopment (*p* < 0.001). In addition, they had higher scores on depressive and anxious symptomatology than parents with typically neurodeveloping children (*p* < 0.0001)	The pandemic caused by COVID-19 has resulted in parents of children with ASD experiencing psychological distress, anxiety and depression

*HADS* Hospital Anxiety and Depression Scale, *SAS* The Self-Rating Anxiety Scale, *PSI-SF* Parent Stress Index Short Form, *GHQ-12* General Health Questionnaire, *DASS-21* Self-Reported Depression, Anxiety and Stress Scale Questionnaire, *CD-RISC 25* Connor-Davidson Resilience Scale, *BFDS* The Brief Family Distress Scale, *FQOL* The Family Quality of Life Scale, *PSS* Parental Stress Scale, *CRS* Coparenting Relationship Scale, *SDQ* Strengths and Difficulties Questionnaire, *ABC* Aberrant Behavior Checklist, *PSQI* Pittsburgh Sleep Quality Index, *BAI* Beck Anxiety Inventory, *PedsQL FIM* Pediatric Quality of Life Inventory Family Impact Module, *CMBP* Special needs children’s Mental and Behavioral Problems, *PMBP* Parents’ Mental and Behavioral Problems Questionnaire, *PSI-SF-15* The Parenting Stress Index-Short Form-15, *MSPSS* Multidimensional Scale of Perceived Social Support, *NEO-FFI* Neo Five-Factor Inventory, *S-AI* The State Anxiety Inventory, *Leiter-R* Leiter International Performance Scale-Revised, *WPPSI-III* Wechsler Preschool And Primary Scale of Intelligence Third Edition, *WISC-IV* Wechsler Intelligence Scale for Children Fourth Edition, *ABAS-II* Adaptive Behavior Assessment System Second Edition, *ADOS-II* Autism Diagnostic Observation Schedule Second Edition, *RBS-R* Repetitive Behavior Scale-Revised, *CBCL* Achenbach Child Behavior Checklist, *PSCQ* Psychological Stress From the Covid-19 Questionnaire, *SCSQ* Simplified coping style questionnaire, *SDS* The Self-Rating Depression Scale, *PSS-10* Perceived Stress Scale, *VABS-II* Vineland Adaptive Behavior Scale-Second Edition, *BL-R* Brunet Lézine- Revised Development Scale, *BECS* Cognitive and Socio-Emotional Evaluation Battery, *PEP-3* Psychoeducational Profile Third Edition, *WPPSI-IV* Wechsler Preschool and Primary Scale of Intelligence Fourth Edition, *WISC-V* Wechsler Intelligence Scale for Children-Fifth Edition, *WAIS-IV* Wechsler Adult Intelligence Scale-Fourth Edition, *K-ABC* Kaufam Assessment Battery for Children, *PHQ-9* The 9-item Patient Health Questionnaire, *GAD-7* The 7-item Generalized Anxiety Disorder Questionnaire, *STAI-6* Short-form of the State- Trait Anxiety Inventory *BRCS* The Brief Resilient Coping Scale

*p < 0.05, **p < 0.01

### Quality assessment

Quality assessment was conducted by using the appropriate respective appraisal tool for analytical cross-sectional studies denominated the Joanna Briggs Institute Critical Appraisal Checklist (JBI) [25]. This evaluation instrument evaluates the quality of the studies according to eight items. The items refer to the clarity of the sample inclusion criteria, identification of confounding factors and strategies to manage them, reliable and valid measurement of the results, etc. In terms of response options, each item can be rated with ''yes'', ''no'', ''unclear'' and ''not applicable''. Studies that score 6 or more times ''yes'' are considered high-quality studies, therefore, those scoring 6 or higher will be part of the present review [9]. According to the criteria of the previously mentioned checklist, eight studies were excluded from this review, because they did not obtain a positive score in 6 or more items. In general, these studies were eliminated, because many of them did not adequately specify the sample inclusion criteria and did not measure the results in a valid and reliable way. On the other hand, some of them did not analyze the confounding factors and the diagnosis of the participants was not clear and had no scientific evidence. This can be seen in more detail in Table 3.

**Table 3:** Quality assessment of the studies after the application of the Joanna Briggs Institute Critical Appraisal Checklist (JBI) [25]

	Item 1	Item 2	Item 3	Item 4	Item 5	Item 6	Item 7	Item 8	Overall appraisal
Alhuzimi [2]	Yes	Yes	Yes	Yes	Yes	Yes	No	Yes	Included study (7/8)
Althiabi [3]	Yes	Yes	Yes	Yes	Yes	Yes	No	Yes	Included study (7/8)
Amorim et al. [5]	Yes	Yes	Yes	Yes	Yes	Yes	No	Yes	Included study (7/8)
Asbury et al. [6]	No	Yes	No	No	No	No	No	No	Excluded study (1/8)
Bentenuto et al. [7]	Yes	Yes	Yes	Yes	Yes	Yes	No	Yes	Included study (7/8)
Berard et al. [8]	Yes	Yes	Yes	Yes	Yes	Yes	No	Yes	Included study (7/8)
Chan and Fung [11]	Yes	Yes	Yes	Yes	Yes	Yes	No	Yes	Included study (7/8)
Chen et al. [12]	Yes	Yes	Yes	Yes	Yes	Yes	No	Yes	Included study (7/8)
Colizzi et al. [13]	No	Yes	No	No	No	No	No	Yes	Excluded study (2/8)
Friesen et al. [18]	Yes	Yes	Yes	Yes	Yes	Yes	No	Yes	Included study (7/8)
Guller et al. [20]	Yes	Yes	Yes	Yes	Yes	Yes	No	Yes	Included study (7/8)
Hosokawa et al. [23]	Yes	Yes	Yes	Yes	Yes	Yes	No	Yes	Included study (7/8)
Huang et al. [24]	No	Yes	No	No	No	No	No	Yes	Excluded study (2/8)
Karahan et al. [26]	Yes	Yes	Yes	No	No	No	No	Yes	Excluded study (4/8)
Kawaoka et al. [28]	Yes	Yes	Yes	Yes	Yes	Yes	No	Yes	Included study (7/8)
Levante et al. [32]	Yes	Yes	Yes	Yes	Yes	Yes	No	Yes	Included study (7/8)
Lim et al. [33]	Yes	Yes	Yes	Yes	Yes	Yes	No	Yes	Included study (7/8)
López-Serrano et al. [34]	Yes	Yes	Yes	Yes	Yes	Yes	Yes	Yes	Included study (8/8)
Lugo-Marín et al. [35]	Yes	Yes	Yes	Yes	No	No	No	Yes	Excluded study (5/8)
Meral [37]	Yes	Yes	Yes	Yes	Yes	Yes	No	Yes	Included study (7/8)
Morris et al. [39]	No	Yes	No	No	No	No	No	Yes	Excluded study (2/8)
Mumbardó-Adam et al. [40]	Yes	Yes	Yes	Yes	Yes	Yes	No	Yes	Included study (7/8)
Mutluer et al. [41]	Yes	Yes	Yes	Yes	Yes	Yes	Yes	No	Included study (7/8)
Pecor et al. [42]	Yes	Yes	Yes	No	Yes	Yes	Yes	Yes	Included study (7/8)
Ren et al. [43]	Yes	Yes	Yes	Yes	No	No	Yes	Yes	Included study (6/8)
Siracusano et al. [45]	Yes	Yes	Yes	Yes	Yes	Yes	Yes	Yes	Included study (8/8)
Vasa et al. [47]	Yes	Yes	Yes	Yes	No	No	No	Yes	Excluded study (5/8)
Wang et al. [49]	Yes	Yes	Yes	Yes	Yes	Yes	Yes	Yes	Included study (8/8)
White et al. [50]	No	Yes	No	No	No	No	No	Yes	Excluded study (2/8)

Items from the Joanna Briggss Institute Critical Appraisal Checklist (JBI) [25]

Item 1: Were the criteria for inclusion in the sample clearly defined?

Item 2: Were the study subjects and the setting described in detail?

Item 3: Was the exposure measured in a valid and reliable way?

Item 4: Were objective, standard criteria used for measurement of the condition?

Item 5: Were confounding factors identified?

Item 6: Were strategies to deal with confounding factors stated?

Item 7: Were the outcomes measured in a valid and reliable way?

Item 8: Was appropriate statistical analysis used?

## Results

A total of 412 studies were found and after removal of 148 duplicates, 264 studies remained. These studies were reviewed for title and abstract, and 200 were excluded, because they did not comply the proposed inclusion and exclusion criteria (see Supplementary Material S1). From the 64 studies that remained in the final full-text eligibility phase, 35 studies were eliminated, because they did not match the following criteria (see Supplementary Material S2): 11 studies were removed, because they did not analyze the psychological, behavioral, and affective-emotional aspects produced by the pandemic in the infant-juvenile population with ASD; 10 studies were eliminated, because they did not represent cross-sectional or longitudinal studies, 6 studies were deleted because they did not include the main population of this review (children and adolescents with ASD or their relatives), 3 studies were not an intervention, and 5 studies were duplicated or not found.

**Fig. 1:**
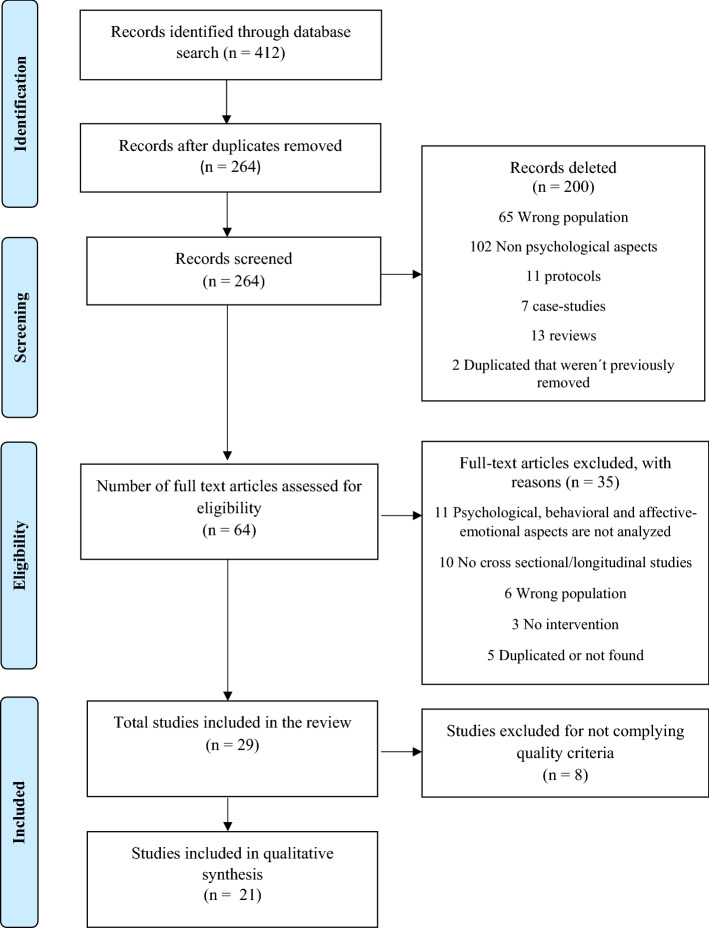
Flowchart of the PRISMA criteria. Source: own elaboration

Finally, the studies included in the final phase for inclusion in this systematic review were 29 articles. However, after analyzing the quality of each study using the Joanna Briggs Institute Critical Appraisal Checklist (JBI) (Jordan et al., 2020), 21 studies were included in the study (see Fig. 1).

### Characteristics of the studies included

The characteristics of the 21 studies included in this review can be seen in detail in Table 2. The participants in the studies were children/adolescents (from 3 to 18 years of age) diagnosed with ASD or their immediate family members, in this case their parents. However, in seven studies, they also included children/adolescents with typical neurodevelopment to be able to compare between clinical and non-clinical samples. The total number of participants in all studies was 13.599. All studies were published between 2020 and 2022 and were conducted in different countries.

In terms of the type of research, all the studies included in the review were cross-sectional and had the objective of analyzing the impact of the pandemic caused by COVID-19 on children/adolescents with ASD, as well as on their family members and associated risk factors. As for the data collection, due to the restrictions imposed by the pandemic, most studies performed online data collection. Participants completed the questionnaires via platforms, social networks or email.

Regarding the instruments used in the research collected, the majority employed validated and standardized like the *Parental Stress Index Short Form (PSI-SF)* [1], the *Hospital Anxiety and Depression Scale (HADS)* [52], the *Generalized Anxiety Disorder (GAD-7)* [46], *the Depression Patient Health Questionnaire (PHQ-9)* [30] and the *Connor Davidson Resilience Scale (CD-RISC 25)* [14]. On the other hand, in some studies, the authors used self-made questionnaires containing items to measure the pre-post COVID impact on both children/adolescents and their family members. The creation of these questionnaires has originated due to the lack of specific instruments to analyze this type of variables. The studies included were categorized under four headings:

#### Anxiety, depression and parental stress

Aspects related to anxiety, depression and parental stress play an essential role during the pandemic. This is mainly due to coping problems, family environment and rigidity in the face of change. On the other hand, the collapse of health care and the reduction of those supports caused family environments to be destabilized, making it all worse.

This is demonstrated by the studies of Alhuzimi [2] or Bentenuto et al. [7], in which they focused on analyzing the changes that occurred in users with ASD during the pandemic with significant results in variables related to parental stress (family environment), as well as in the symptomatology of the disorder itself (stereotypies and behaviors characteristic of ASD). One of the aspects detected as crucial was the lack of support and ineffectiveness of the services compared to situations prior to confinement, leading to a considerable increase in parental stress levels of approximately 43% and the development of external behaviors of the children that generated predictors of behavioral changes.

Others such as Lim et al. [33], Friesen et al. [18], Ren et al. [43] or Wang et al. [49] highlighted the way of coping with this perceived stress as key on the part of the relatives and the type of coping during the adaptation process (resilience as a determining variable) and introduced important factors such as the gender of the caregiver (in some cases greater in women than in men), the purchasing power could alleviate stressful situations for the parents and even the level of educational training they had received during their educational stage, to determine the tools they had available to deal with the problem. Such an attitude, as presented by Althiabi [3], is the result of a significant negative correlation between the anxiety variables and the mental health status of the parents themselves, in which the latter deteriorated due to the care problems that occurred during the pandemic and the confinement.

If we refer to variables that acquire great importance, such as quality of life and family happiness, Meral [37] highlighted significant negative correlations between parental distress and family quality through the analysis of the impact of confinement. These may be important variables in those family units that present greater parental distress and, therefore, a possible lower quality of life (family environment), being especially negative towards children because of the consequences produced in their quality of life, with or without a normotypical development. On the other hand, Pecor et al. [42] also analyzed the quality of life of caregivers of children with ASD/ADHD, reaching the conclusion of a lower quality of life for these caregivers compared to caregivers of normotypical children, suffering greater anxiety, stress, depression and emotional dysregulation during confinement.

Chen et al. [12], through the evaluation of mental health in parents of children with special needs, highlighted not only the great difference according to the characteristics of the children, but also the high probability of presenting mental health problems in families with children with ASD, compared to others such as visual or hearing impairment. Some of the aspects analyzed were behavioral problems, parent–child interaction, family/environment/specific support.

In addition to those described above, health problems associated with the pandemic in clinical and non-clinical populations, as in the study by Chan and Fung [11], made it possible to evaluate changes in depressive symptomatology through the creation of a questionnaire *(PHQ-9)*, associating significantly high levels comparing families with clinical and non-clinical populations. These health problems being one of the major problems of all families regardless of their pandemic situation.

#### Anxiety in children with ASD

Regarding anxiety in children with ASD during the pandemic, we can highlight the research conducted by Amorim et al. [5], whose objective was to discover how children with ASD and their families experience social isolation during the quarantine. In this research, a cross-sectional analytical study was conducted involving parents of children diagnosed with ASD and parents of children without any clinical diagnosis (control group). Due to this investigation, it was observed that parents of children with ASD reported significant changes in their children's behavior, whereas parents of children in the control group reported no changes in behavior. The differences between the two groups were statistically significant, with anxiety being one of the symptoms experienced more frequently by children with ASD compared to other types of behaviors. Furthermore, in this research, it was observed that children with ASD who did not maintain routines had significantly higher levels of anxiety compared to those who did.

#### Behavioral change in children with ASD

Considering the behavioral changes experienced by children with ASD during the pandemic, it is important to highlight the research conducted by Guller et al. [20] whose objective was to analyze what were the emotional and behavioral changes experienced by children with neurodevelopmental disorders during the pandemic. Through this study, it was observed that 33.4% of parents reported behavioral problems during the pandemic. Hyperactivity and repetitive/stereotyped behaviors were the problems most frequently identified by parents. Specifically, in children with ASD, 50.4% experienced repetitive/stereotyped behaviors and 46.6% experienced hyperactivity. Therefore, through this research, we can observe a significant increase in behavioral problems associated with children with neurodevelopmental disorders compared to the previous era.

Other researchers such as Mutluer et al. [41] conducted a study in which they explored the effects of the pandemic on children/adolescents diagnosed with ASD. Thus, 55% of parents reported that their child became more aggressive, 26% reported that their child's tics increased and new ones appeared, and finally, 29% of parents reported a deterioration in their child's communication skills. In addition, this research showed that stereotypical hat behavior and hyperactivity increased compared to the time before the onset of the virus. In addition, social isolation and irritability were other variables that were increased in this population.

Like the aforementioned results, Hosokawa et al. [23] investigated differences in behavioral changes in children diagnosed with ASD and undiagnosed children and adolescents during the pandemic. This work indicated that a higher percentage of children with ASD (45.2%) felt frustrated due to the change in their routines compared to children in the control group (31.0%). An increase in restricted and repetitive behaviors was also reported in the ASD group.

In another study by López-Serrano et al. [34] whose aim was to explore the psychological impact of seclusion on child and adolescent patients in a mental health center in Barcelona, the main findings showed a higher frequency of self-injury and regressive behaviors in children/adolescents with ADHD, ASD and Anxiety Disorders compared to those with Affective Disorders. On the other hand, children/adolescents with ASD presented higher scores in obsessive–compulsive symptoms and stereotyped movements. Through this project, it was also observed that having close relatives and loss of income correlated with a higher level of parental stress and a greater impact on the symptoms of children/adolescents. Along the same lines, in the studies by Levante et al. [32] and Kawaoka et al. [28], the results showed that ASD patients had significantly elevated scores on externalizing and aggressive behaviors.

In conclusion, the restrictions caused by the pandemic originated by COVID-19 have been the cause of the psychological impact that has occurred in the infant-juvenile population with developmental disorders, as well as in their immediate family members.

#### The positive effects of the pandemic

Although most studies have shown that the pandemic caused by COVID-19 has had a negative impact on different aspects related to the symptomatology of children with ASD or the quality of life of their families, there are three studies included in this review that demonstrate the opposite.

First, the results obtained from the research conducted by Berard et al. [8] showed that approximately half of the parents reported no changes in their child's sleep, communication skills, or stereotyped behaviors. In addition, 28.8% of the sample reported an improvement in their children's social communication. This improvement in children's social communication was related to the maintenance and continuation of therapeutic interventions during confinement.

Consistent with the above, the study by Mumbardó-Adam et al. [40] also found positive results in the functionality of children with ASD during the pandemic. The aim of this study was to learn about parents' management of the quarantine period to meet the needs of their children. It was observed that 14.9% of the children obtained higher levels of autonomy in self-care (personal hygiene, feeding, dressing…) during the quarantine period. In addition, 19.2% improved their communication skills and 27.7% participated more frequently in the family environment (setting the table, deciding the type of activities to do…) and also, families reported that their children were happier, and calmer compared to the previous period.

Finally, the aim of the third study done by Siracusano et al. [45] was to investigate in a sample of autistic individuals, any changes in adaptive functioning and in repetitive and behavioral problems (internalizing and externalizing), appeared after mandatory home confinement, through the comparison of data collected during the pandemic with assessments made before the COVID-19 outbreak. It was observed that the group of preschool children obtained an improvement in all domains of their adaptive functioning, except for the social area. These children, however, showed an increase in communication, academic skills, and self-care.

## Discussion

This systematic review has examined the consequences that the pandemic caused by COVID-19 has had on the child and adolescent population with ASD, as well as on their immediate family members. Consequently, the main objective of all the studies included in this review was to analyze and evaluate the impact of the pandemic on the mental health of these children/adolescents and their family environment. In addition, all studies included in this review were of high quality, because they obtained a score of 6 or higher on the Joanna Briggs Institute Critical Appraisal Checklist (JBI) [25].

Moreover, several studies [10, 22, 31] state that parents of children with ASD presented increased levels of stress, as well as depressive and anxious symptoms due to the high caregiving demands required by children with this type of disorder. Moreover, with the emergence of the pandemic caused by COVID-19, these symptoms in parents have been exacerbated.

Through the exhaustive analysis of each of the studies included in the review, it has been possible to observe a significant decrease in the psychological well-being of parents of children with ASD (Alhuzimi 2020) [7]. More specifically, in the research done by Guller et al. [20], it was observed that sleep, emotional and behavioral problems of children with ASD have had a negative impact on the mood of their parents, increasing their levels of anxiety and depression. On the other hand, in another study carried by López-Serrano et al. [34] it has been shown that, in addition to the aforementioned, the cognitive inflexibility of children and their constant worries related to death by COVID-19 have increased the stress levels of their parents. Furthermore, Althiabi [3] revealed that the parents of the children/adolescents with ASD felt more tired than usual, as they had to dedicate more time to their children during the pandemic and often assumed the role of teachers. Along the same lines, the lack of support and ineffectiveness of health services as well as school services have led to increased levels of parental stress (Alhuzimi 2020) [3, 43]. In general, it can be observed how each research provides relevant information about the different factors that have played an important role in the increase of parental stress and in their adaptation to this new situation produced by the pandemic.

On the other hand, even though most of the studies included in this review show that the appearance of COVID-19 increased the severity of the symptomatology of children with autism, causing at the same time higher levels of anxiety and depression in their relatives, there are also investigations that show the opposite. One of these investigations is the one conducted by Berard et al. [8], whose main objective was to examine whether changes in sleep, feeding, communication or stereotyped behavior in children with ASD have occurred during seclusion. The results showed that half of the parents reported no changes in their children's sleep, communication skills, or stereotyped behaviors during confinement. Similarly, in another study by Mumbardó-Adam et al. [40] involving 47 parents of children with ASD, it was observed that 14.9% of the children had increased levels of autonomy in relation to self-care during the quarantine period. At the same time, 19.2% of the children improved their communication skills and 27.7% participated more frequently in the family context.

Finally, this systematic review shows how children/adolescents with ASD and their families have coped with the pandemic caused by COVID-19. It has been observed throughout the study that most families experienced this situation in a stressful way, due to severe changes in routines, limited access to socio-health/educational resources and the high care demands required by these children/adolescents.

### Limitations

This review is not without limitations. Firstly, the small number of validated and standardized instruments for measuring the psychological impact of the pandemic limits the validity of the results obtained in some studies, with some of the studies losing out on aspects such as quality analysis. Likewise, the lack of homogeneity among the existing instruments makes comparison between the different studies difficult, leading to possible biases in terms of variability and approaches depending on the tool used.

Secondly, the low number of participants in some of them makes it difficult to generalize the results. This is possibly due to difficulties with the sample. Thirdly, all the studies included were cross-sectional, which precludes making causal inferences. In addition, in most of the investigations, the instruments and questionnaires were administered in greater numbers to family members than to people with ASD, so that the results obtained have focused more on the opinions and perceptions of parents than on the users with ASD themselves. Additionally, another limitation of this review is that, despite the general measures adopted in all countries, such as school closures, home quarantine and social isolation, a more detailed analysis of exactly how this situation was managed in each country and the specific period of confinement adopted by the different countries should be done. However, it has been seen that, in general, the restrictions imposed by governments around the world have had a negative psychological impact on the child-adolescent population. Finally, most of the studies collected data online, which makes people with limited access to the Internet less involved in the research.

### Implications for future research and practice

It would be desirable for future research to create or use validated and standardized instruments specifically to measure the psychological consequences and impact of COVID-19 in both child and adolescent populations with neurodevelopmental disorders and in the adult population. Another possible aspect is the generation of intervention protocols or data collection for the systematization and replication of results. Long-term monitoring of the effects of the pandemic in these populations should also be carried out. In addition, it would be interesting to carry out specific interventions to address and treat the problems caused by the pandemic in the future.

## Conclusions

This systematic review provided relevant information on the psychological impact of the pandemic caused by COVID-19 on children/adolescents diagnosed with autism and their family environment. Through the studies analyzed throughout the present work, it is observed that most children with ASD, as well as their families, experienced certain difficulties, and challenges during the quarantine.

The findings of the different studies included in this review indicate that parents' anxiety, depression, and stress levels increased due to the behavioral alterations of children with ASD during confinement. Likewise, changes in routine, school closure and social isolation have led to an increase in children's stereotyped and aggressive behaviors, as well as increased hyperactivity, frustration, and irritability. Therefore, it has been seen, in most studies, that the pandemic has generated a great negative psychological impact on this population. However, there is also research showing that children/adolescents with ASD have coped positively with the quarantine, increasing their levels of autonomy and self-care, as well as their communication skills.

In conclusion, the pandemic has had a significant impact on the lives of these children/adolescents and on their family context to provide them with adequate supports that fit their needs. Therefore, the effects on these populations should be monitored over the long term to ensure that future interventions effectively address this issue.

### Supplementary Information

Below is the link to the electronic supplementary material.

Supplementary file1 (DOCX 53 KB)

## Data Availability

Data sharing not applicable to this article as no datasets were generated or analyzed during the current study.
