# Supplementary material for: The psychological impact of the COVID-19 pandemic on children/adolescents with ASD and their family environment: a systematic review
Source: Eur Child Adolesc Psychiatry. 2023 Feb 9;33(1):203–28. doi: 10.1007/s00787-023-02151-6 (PMC9909131; doi:10.1007/s00787-023-02151-6)
Supplement: Supplementary file 1 — Supplementary file1 (DOCX 53 KB) [file 787_2023_2151_MOESM1_ESM.docx]

S1: Basic data of the studies excluded in the screening phase

| **Reason** | **Títle** | **Authors (year)** |
| --- | --- | --- |
| **Wrong Population (N=65)** | Commentary: Challenges and opportunities in autism assessment—A commentary on Kanne and Bishop (2020) | MacLachlan, M. (2021) |
|  | Moral courage, moral sensitivity and safe nursing care in nurses caring of patients with COVID-19 | Khodaveisi et al. (2021) |
|  | Risks and Protective Factors Associated With Mental Health Symptoms During COVID-19 Home Confinement in Italian Children and Adolescents: The #Understandingkids Study | Oliva et al. (2021) |
|  | The Impact of COVID-19 on Anxiety and Worries for Families of Individuals with Special Education Needs and Disabilities in the UK | Sideropoulos et al. (2021) |
|  | Zika Brazilian Cohorts (ZBC) Consortium: Protocol for an Individual Participant Data Meta-Analysis of Congenital Zika Syndrome after Maternal Exposure during Pregnancy | ZBC-Consortium et al. (2021) |
|  | Use of Telehealth in Fellowship-Affiliated Developmental Behavioral Pediatric Practices During the COVID-19 Pandemic | Wallis et al. (2021) |
|  | Scoping exercise to develop a storybook to support children's education during the COVID-19 pandemic | Syeda et al. (2021) |
|  | Another vision from the coronavirus health crisis in Spain: The perspective from the Plena inclusión developmental disabilities associative movement | Galván (2021) |
|  | COVID-19 during Pregnancy and Postpartum:: I) Pathobiology of Severe Acute Respiratory Syndrome Coronavirus-2 (SARS-CoV-2) at Maternal-Fetal Interface | Naidu et al. (2020) |
|  | Tele-practice for children and young people with communication disabilities: Employing the COM-B model to review the intervention literature and inform guidance for practitioners | Law et al. (2021) |
|  | Developing and enhancing adherence to a telehealth ABA parent training curriculum for caregivers of children with autism | Yi & Dixon (2021) |
|  | The mental health crises of the families of COVID-19 victims: a qualitative study | Mohammadi et al. (2021) |
|  | COVID-19 Morbidity Among Individuals with Autistic Spectrum Disorder: A Matched Controlled Population-Based Study | Krieger et al. (2021) |
|  | Impact of COVID-19: Considerations for individuals with developmental disabilities across major life domains | Sheppard-Jones et al. (2021) |
|  | Caregivers' perception of the caring challenges in coronavirus crisis (COVID-19): a qualitative study | Mohammadi et al. (2021) |
|  | Assessment of Maternal and Neonatal SARS-CoV-2 Viral Load, Transplacental Antibody Transfer, and Placental Pathology in Pregnancies During the COVID-19 Pandemic | Edlow et al. (2020) |
|  | Healthcare-Associated Legionnaires' Disease, Europe, 2008-2017 | Beaute et al. (2020) |
|  | Risk of neuropsychiatric disorders in offspring of COVID-19-infected pregnant women and nutritional intervention | Hashimoto (2021) |
|  | Covid‐19‐related psychiatric impact on italian adolescent population: A cross‐sectional cohort study | Mensi et al. (2021) |
|  | Teaching Small Talk: Increasing On-Topic Conversational Exchanges in College Students with Intellectual and Developmental Disabilities Using Remote Audio Coaching | Joseph et al. (2021) |
|  | Rapid establishment of a COVID-19 perinatal biorepository: early lessons from the first 100 women enrolled | Shook et al. (2020) |
|  | Using real patients in e-learning: case-based online training in child and adolescent psychiatry | Taurines et al. (2020) |
|  | Psycho-social factors associated with mental resilience in the Corona lockdown | Veer et al. (2021) |
|  | Vulnerable and Forgotten: The Impact of the COVID-19 Pandemic on Autism Special Schools in England | Crane et al. (2021) |
|  | A peer interview qualitative study exploring support for carers of people with comorbid autism and eating disorders | Kinnaird et al. (2021) |
|  | Evaluating Clinical Course and Risk Factors of Infection and Demographic Characteristics of Pregnant Women with COVID-19 in Hamadan Province, West of Iran | Sattari et al. (2020) |
|  | Clinical management of individuals with Intellectual Disability: The outbreak of Covid-19 pandemic as experienced in a clinical and research center Research in Developmental Disabilities | Buono et al. (2021) |
|  | A Proposed Process for Risk Mitigation During the COVID-19 Pandemic | Cox et al. (2020) |
|  | Effective strategies for managing covid-19 emergency restrictions for adults with severe asd in a daycare center in Italy | Brondino et al. (2020) |
|  | Effects of COVID-19 Lockdown on the Emotional and Behavioral Profiles of Preschool Italian Children with and without Familial Risk for Neurodevelopmental Disorders | Cantiani et al. (2021) |
|  | Prevalence and Associated Factors of Emotional and Behavioural Difficulties during COVID-19 Pandemic in Children with Neurodevelopmental Disorders | Nonweiler et al. (2020) |
|  | Cognitive Behavioral Therapy for Autism Spectrum Disorders: A Systematic Review | Wang et al. (2021) |
|  | G2P[4] rotavirus outbreak in Belu, East Nusa Tenggara Province, Indonesia, 2018 | Utsumi et al. (2020) |
|  | Availability of Services and Caregiver Burden: Supporting Individuals With Neurogenetic Conditions During the COVID-19 Pandemic | Kowanda et al. (2021) |
|  | The effects of transition to technician‐delivered telehealth aba treatment during the COVID‐19 crisis: A preliminary analysis | Pollard et al. (2021) |
|  | Maternal nutrients and effects of gestational COVID-19 infection on fetal brain development | Hoffman et al. (2021) |
|  | A Qualitative Study of Child and Adolescent Mental Health during the COVID-19 Pandemic in Ireland | O' Sullivan et al. (2021) |
|  | Three-Year Clinical Follow-Up of Children Intrauterine Exposed to Zika Virus | Gazeta et al. (2021) |
|  | Remote learning for children with Special Education Needs in the era of COVID-19: Beyond tele-conferencing sessions | Aloizou et al. (2021) |
|  | Clinical intervention with autistic adolescents and adults during the first Two months of the COVID-19 pandemic: Experiences of clinicians and their clients | Southey & Stoddart (2021) |
|  | WSU ROAR and ROAR Online! Program Description and COVID-19 Response | McMahon et al. (2021) |
|  | In utero Zika virus exposure and neurodevelopment at 24months in toddlers normocephalic at birth: a cohort study | Grant et al. (2021) |
|  | Impact of the COVID-19 Italian Lockdown on the Physiological and Psychological Well-Being of Children with Fragile X Syndrome and Their Families | Di Giorgio et al. (2021) |
|  | The Efficacy of WeChat-Based Parenting Training on the Psychological Well-being of Mothers With Children With Autism During the COVID-19 Pandemic: Quasi-Experimental Study | Liu et al. (2021) |
|  | Health Professionals' Perception of Psychological Safety in Patients with Coronavirus (COVID-19) | Mohammadi et al. (2020) |
|  | Implementing group parent training in telepsychology: Lessons learned during the COVID-19 pandemic | Fogler et al. (2020) |
|  | COVID-19-related prescribing challenge in intellectual disability | Rauf et al. (2021) |
|  | Rapid Conversion from Clinic to Telehealth Behavioral Services During the COVID-19 Pandemic | Crockett et al. (2020) |
|  | Syndemic Conditions, Sexual Risk Behavior, and HIV Infection Among Men Who Have Sex with Men in Taiwan | Chuang et al. (2021) |
|  | Autism Spectrum Disorder and COVID-19: Helping Caregivers Navigate the Pandemic | Lim et al. (2020) |
|  | Impact of COVID-19 outbreak on mental health and perceived strain among caregivers tending children with special needs | Dhiman et al. (2020) |
|  | Parents’ perceptions on physical activity for their children with autism spectrum disorders during the novel coronavirus outbreak | Esentürk (2020) |
|  | Remote learning during COVID-19: Examining school practices, service continuation, and difficulties for adolescents with and without attention-deficit/hyperactivity disorder | Becker et al. (2020) |
|  | Examining the impact of COVID-19 in ethnically diverse families with young children with intellectual and developmental disabilities | Neece et al. (2020) |
|  | COVID-19 remote learning experiences of youth with neurodevelopmental disorders in rural Appalachia | McFayden et al. (2021) |
|  | Home education for children with autism spectrum disorder during the COVID-19 pandemic: Indonesian mothers experience | Daulay (2021) |
|  | COVID-19-related fear and stress among individuals who experienced child abuse: The mediating effect of complex posttraumatic stress disorder | Tsur & Abu-Raiya (2020) |
|  | Kawasaki-like disease among Italian children in the COVID-19 era | Kuo (2020) |
|  | Mapping routine measles vaccination in low- and middle-income countries | Sbarra et al. (2021) |
|  | Prospects for improving future mental health of children through prenatal maternal micronutrient supplementation in China | Li & Freedman (2020) |
|  | Physical activity moderates the association between parenting stress and quality of life in working mothers during the COVID-19 pandemic | Limbers et al. (2020) |
|  | Immunopsychiatry and SARS-CoV-2 pandemic: Links and possible consequences | Daaboul et al. (2021) |
|  | Caring for the Most Vulnerable: A Model for Managing Maladaptive Behavior in Children with Mental Special Needs During the COVID-19 Pandemic | Dursun et al. (2021) |
|  | The Disproportionate Burden of the COVID-19 Pandemic Among Pregnant Black Women | Gur et al. (2020) |
|  | Prevalence and predictors of psychological response during immediate COVID-19 pandemic | Cansel et al. (2021) |
| **Non-Psychological aspects**  **(N=102)** | Tweet Topics and Sentiments Relating to COVID-19 Vaccination Among Australian Twitter Users: Machine Learning Analysis | Kwok et al. (2021) |
|  | Development, Feasibility, and Acceptability of a Nationally Relevant Parent Training to Improve Service Access During the Transition to Adulthood for Youth with ASD | Taylor et al. (2022) |
|  | Global Measles Epidemic Risk: Current Perspectives on the Growing Need for Implementing Digital Communication Strategies | Bozzola et al. (2020) |
|  | Moving Toward Telehealth Surveillance Services for Toddlers at Risk for Autism During the COVID-19 Pandemic | Conti et al. (2020) |
|  | Debate: Remote learning during covid‐19 for children with high functioning autism spectrum disorder | Reicher (2020) |
|  | Creating an automated health attestation system during the COVID-19 pandemic with Microsoft 365 | Dubuque et al. (2020) |
|  | Everyday challenges and caring possibilities for children and adolescents with autistic spectrum disorder (asd) in the face of covid-19 | Fernandes et al. (2021) |
|  | Autism Spectrum Self-Stimulatory Behaviors Classification Using Explainable Temporal Coherency Deep Features and SVM Classifier | Liang et al. (2021) |
|  | A Qualitative Study On People's Experiences Of Covid-19 Media Literacy | Jormand et al. (2021) |
|  | COVID-19 health and social care access for autistic people: European policy review | Oakley et al. (2021) |
|  | Potential health benefits of sustained air quality improvements in New York City: A simulation based on air pollution levels during the COVID-19 shutdown | Perera et al. (2021) |
|  | COVID-19 and behaviors in children with autism spectrum disorder: Disparities by income and food security status | Panjwani et al. (2021) |
|  | Autism and access to care during the COVID-19 crisis | Nadler et al. (2021) |
|  | Obsessive compulsive disorder and obsessive compulsive personality disorder and the criminal law | Freckelton (2020) |
|  | Fifteen-minute consultation: Vaccine-hesitant parents | Bedford & Elliman (2020) |
|  | COVID‐19 and autism research: Perspectives from around the globe: Challenges of conducting longitudinal MRI studies in the time of COVID-19 | Wu Nordahl (2020) |
|  | The (almost) impossible profession: Face-to-face child psychotherapy during the Covid-19 outbreak | Shulman (2020) |
|  | The digital divide in technologies for autism: Feasibility considerations for low- and middle-income countries | Kumm et al. (2021) |
|  | Real-Time Telehealth Treatment Team Consultation for Self-Injury by Individuals with Autism Spectrum Disorder | Singh et al. (2021) |
|  | Association between Viral Infections and Risk of Autistic Disorder: An Overview | Shuid et al. (2021) |
|  | Anything but the phone!': Communication mode preferences in the autism community | Howard & Sedgewick (2021) |
|  | Enhancing Communication Skills of Individuals With Autism Spectrum Disorders While Maintaining Social Distancing Using Two Tele-Operated Robots | Kumazaki et al. (2021) |
|  | Masking Emotions: Face Masks Impair How We Read Emotions | Gori et al. (2021) |
|  | Increasing passive compliance to wearing a facemask in children with autism spectrum disorder | Lillie et al. (2021) |
|  | COVID‐19 and autism research: Perspectives from around the globe | Amaral & de Vries (2020) |
|  | The Financial Situation of Families and the Quality of Life and Coping with Stress of Children with ASD during the SARS-CoV-2 Pandemic | Gagat-Matula (2021) |
|  | Coparenting autistic children during COVID-19: Emerging insights from practice | Southey et al. (2021) |
|  | Neurologic Care of COVID-19 in Children | Boronat (2021) |
|  | COVID‐19 and autism research: Perspectives from around the globe: COVID-19 in Africa: Magnifying pre-existing digital and socio-economic disparities, but with glimmers of hope | de Vries et al. (2020) |
|  | Altered patterns of brain dynamics linked with body mass index in youth with autism | Kupis et al. (2021) |
|  | IoT-fog-cloud based architecture for smart systems: Prototypes of autism and COVID-19 monitoring systems | Kallel et al. (2021) |
|  | Climate change, environment pollution, COVID-19 pandemic and mental health | Marazziti et al. (2021) |
|  | Vaccine hesitancy and reported non-vaccination in an Irish pediatric outpatient population | Whelan et al. (2021) |
|  | Autism, Therapy and COVID-19 | Sergi et al. (2021) |
|  | Pragmatic adaptations of telehealth-delivered caregiver coaching for children with autism in the context of COVID-19: Perspectives from the United States and South Africa | Franz et al. (2021) |
|  | The Pediatric Neurology 2020 Research Workforce Survey: Optimism in a Time of Challenge | Bonkowsky et al. (2021) |
|  | Editorial perspective: Perils and promise for child and adolescent sleep and associated psychopathology during the COVID‐19 pandemic | Becker & Gregory (2020) |
|  | COVID‐19 and autism research: Perspectives from around the globe: Considerations for conducting telehealth research with Latino children and adults with ASD in the US | Toth et al. (2020) |
|  | Establishment of a pediatric COVID-19 biorepository: unique considerations and opportunities for studying the impact of the COVID-19 pandemic on children | Lima et al. (2020) |
|  | Influence of ideational praxis on the development of play and adaptive behavior of children with autism spectrum disorder: A comparative analysis | Serrada-Tejeda et al. (2021) |
|  | Imagination for two' child psychotherapy during coronavirus outbreak: Building a space for play when space collapses | Shulman & Saroff (2020) |
|  | Future perspectives of robot psychiatry: can communication robots assist psychiatric evaluation in the COVID-19 pandemic era? | Yoshikawa et al. (2021) |
|  | Feasibility and Acceptability of a Synchronous Online Parent-Mediated Early Intervention for Children with Autism in a Low Resource Setting During COVID-19 Pandemic | Sengupta et al. (2021) |
|  | Rural Disparities in Early Childhood Well Child Visit Attendance | DeGuzman et al. (2021) |
|  | Naturalistic communication training for early intervention providers and latinx parents of children with signs of autism | Gevarter et al. (2021) |
|  | The social patterning of autism diagnoses reversed in California between 1992 and 2018 | Winter et al. (2020) |
|  | The novel Coronavirus (COVID-19) outbreak: Physical inactivity and children with Autism Spectrum Disorders | Yarımkaya & Esentürk (2020) |
|  | Telehealth mask wearing training for children with autism during the COVID‐19 pandemic | Sivaraman et al. (2021) |
|  | From Helpless to Hero: Promoting Values-Based Behavior and Positive Family Interaction in the Midst of COVID-19 | Szabo et al. (2020) |
|  | How to' operate a pediatric neuropsychology practice during the COVID-19 pandemic: Real tips from one practice’s experience | Loman et al. (2021) |
|  | Chronotypes and trauma reactions in children with adhd in home confinement of covid-19: Full mediation effect of sleep problems | Çetin et al. (2020) |
|  | Prevalence of overweight and obesity in children and adolescents with intellectual disabilities in china | Yuan et al. (2021) |
|  | Using telehealth to provide outpatient follow‐up to children with avoidant/restrictive food intake disorder | Peterson et al. (2021) |
|  | An analysis of a system under pandemic conditions | Oblak (2021) |
|  | Characterizing available tools for synchronous virtual assessment of toddlers with suspected autism spectrum disorder: A brief report | Berger et al. (2021) |
|  | Food insecurity in the households of children with autism spectrum disorders and intellectual disabilities in the United States: Analysis of the National Survey of Children's Health Data 2016-2018 | Karpur et al. (2021) |
|  | Parental attitudes and decisions regarding MMR vaccination during an outbreak of measles among an undervaccinated Somali community in Minnesota | Christianson et al. (2020) |
|  | The public health crisis of underimmunisation: a global plan of action | Gostin et al. (2020) |
|  | Global health disparities in vulnerable populations of psychiatric patients during the COVID-19 pandemic | Diaz et al. (2021) |
|  | Functional assessment and function-based treatment delivered via telehealth: A brief summary | Schieltz & Wacker (2020) |
|  | Childhood vaccination as a protective factor for developmental psychopathology | Kim et al. (2020) |
|  | Taking action: 18 simple strategies for supporting children with autism during the covid-19 pandemic | Tarbox et al. (2020) |
|  | Maternal Immune Activation by Poly I:C as a preclinical Model for Neurodevelopmental Disorders: A focus on Autism and Schizophrenia | Haddad et al. (2020) |
|  | Use of the tele-asd-peds for autism evaluations in response to covid-19: Preliminary outcomes and clinician acceptability | Wagner et al. (2020) |
|  | COVID‐19 and autism research: Perspectives from around the globe: The effect of the COVID-19 pandemic on autism research in Uganda | Mwesige (2020) |
|  | This may be a really good opportunity to make the world a more autism friendly place & rdquo;: Professionals & rsquo; perspectives on the effects of COVID-19 on autistic individuals | Spain et al. (2021) |
|  | The relationship between chronotype, sleep, and autism symptom severity in children with ASD in COVID-19 home confinement period | Türkoğlu et al. (2020) |
|  | Sleep disorders reveal distress among children and adolescents during the Covid-19 first wave: results of a large web-based Italian survey | Dondi et al. (2021) |
|  | Short report on research trends during the COVID-19 pandemic and use of telehealth interventions and remote brain research in children with autism spectrum disorder | Su et al. (2021) |
|  | Your country is your routine: The evacuation, quarantine, and management of behavioral problems of a child with autism during COVID-19 pandemic | Turan et al. (2020) |
|  | Evaluating the cool versus not cool procedure via telehealth | Cihon et al. (2021) |
|  | Telehealth parent coaching to improve daily living skills for children with ASD | Gerow et al. (2021) |
|  | Online training for physical activity practitioners on evidence-based practices for clients with autism | McNamara et al. (2020) |
|  | Sexual Health in Child and Adolescent Psychiatry: Multi-Site Implementation Through Synchronized Videoconferencing of an Educational Resource Using Standardized Patients | Drozdowicz et al. (2020) |
|  | Transformative learning in early-career child and adolescent psychiatry in the pandemic | Kaku et al. (2021) |
|  | Distance Special Education Delivery by Social Robots | Lytridis et al. (2020) |
|  | Interpreters' knowledge and perceptions of childhood vaccines: Effect of an educational session | Madlon-Kay & Smith (2020) |
|  | Alcohol and Tobacco use While Breastfeeding and Risk of Autism Spectrum Disorder or Attention Deficit/Hyperactivity Disorder | Gibson & Porter (2022) |
|  | Use of the brief observation of symptoms of autism (bosa) as a new clinical approach to assessing patients with suspected spectrum disorder during the COVID-19 pandemic | Rynkiewicz et al. (2020) |
|  | COVID‐19 and autism research: Perspectives from around the globe: COVID-19 pandemic offers unique challenges and opportunities that transcend borders | Dawson et al. (2020) |
|  | Developing remote delivery of language and cognitive training for use with children with autism: A technological report | Belisle et al. (2021) |
|  | Benefits, burden, and COVID-19: A response to Dutheil et al (2020) | Rubenstein et al. (2021) |
|  | Transitioning to telemedicine during covid-19: Impact on perceptions and use of telemedicine procedures for the diagnosis of autism in toddlers | Wagner et al. (2021) |
|  | Thriving in the new normal: How COVID-19 has affected alternative learners and their families and implementing effective, creative therapeutic interventions | Saline (2021) |
|  | COVID‐19 and autism research: Perspectives from around the globe: The need of autism research using robotics for combating COVID-19 | Kumazaki et al. (2020) |
|  | The pathophysiology of SARS-CoV-2: A suggested model and therapeutic approach | Morris et al. (2020) |
|  | The possibility and importance of immersive technologies during COVID-19 for autistic people | Newbutt et al. (2020) |
|  | COVID‐19 and autism research: Perspectives from around the globe: Research in the time of COVID-19: A view from India | Divan et al. (2020) |
|  | Psychiatric disorders in children and adolescents during the COVID-19 pandemic | Palacio-Ortiz et al. (2020) |
|  | Diagnostic evaluations of autism spectrum disorder during the covid-19 pandemic | Jang et al. (2021) |
|  | Transforming pediatric neuropsychology through video-based teleneuropsychology: An innovative private practice model pre-COVID-19 | Salinas et al. (2020) |
|  | Reply to: Another vision from the coronavirus health crisis in Spain: The perspective from the Plena inclusión developmental disabilities associative movement | Arango (2021) |
|  | Editorial: Mental health and schools: Has the time arrived? | Pumariega (2021) |
|  | Recommendations for the Biden-Harris administration A statement of priorities from the society for developmental and behavioral pediatrics | Weitzman et al. (2021) |
|  | Tolerance of face coverings for children with autism spectrum disorder | Halbur et al. (2021) |
|  | Changes in access to educational and healthcare services for individuals with intellectual and developmental disabilities during covid‐19 restrictions | Jeste et al. (2020) |
|  | Big data Big problem? | Harrop et al. (2021) |
|  | Using Hybrid Telepractice for Supporting Parents of Children with ASD during the COVID-19 Lockdown: A Feasibility Study in Iran | Samadi et al. (2020) |
|  | Provision of speech-language pathology services for the treatment of speech and language disorders in children during the COVID-19 pandemic: Problems, concerns, and solutions | Tohidast et al. (2020) |
|  | Transition of a Judo Program from In-Person to Remote Delivery During COVID-19 for Youth with Autism Spectrum Disorder | Garcia et al. (2021) |
|  | Connected Play in Virtual Worlds: Communication and Control Mechanisms in Virtual Worlds for Children and Adolescents | Du et al. (2021) |
|  | Randomized controlled trial of a video gaming-based social skills program for children on the autism spectrum | Beaumont et al. (2021) |
| **Protocols**  **(N=11)** | Children's Mental Health in the Time of COVID-19: How Things Stand and the Aftermath | Ramadhan et al. (2020) |
|  | Phase 2 and Later of COVID-19 Lockdown: Is it Possible to Perform Remote Diagnosis and Intervention for Autism Spectrum Disorder? An Online-Mediated Approach | Narzisi (2020) |
|  | Autism spectrum condition and COVID-19: Issues and chances | Narzisi (2020) |
|  | Maintaining Treatment Integrity in the Face of Crisis: A Treatment Selection Model for Transitioning Direct ABA Services to Telehealth | Rodriguez (2020) |
|  | Failure of Healthcare Provision for Attention-Deficit/Hyperactivity Disorder in the United Kingdom: A Consensus Statement | Young et al. (2021) |
|  | Creating and Sharing Digital ABA Instructional Activities: A Practical Tutorial | Mattson et al. (2020) |
|  | Introduction to the special issue: Addressing health disparities in pediatric psychology | Valrie et al. (2020) |
|  | School's Out for COVID-19: 50 Ways BCBA Trainees in Special Education Settings Can Accrue Independent Fieldwork Experience Hours During the Pandemic | Fronapfel & Demchak (2020) |
|  | Enhancing early detection of neurological and developmental disorders and provision of intervention in low-resource settings in Uttar Pradesh, India: Study protocol of the G.A.N.E.S.H. programme | Toldo et al. (2020) |
|  | Reopening the Doors to Center-Based ABA Services: Clinical and Safety Protocols During COVID-19 | Kornack et al. (2020) |
|  | Protocol for a randomized pilot study (FIRST STEPS): implementation of the Incredible Years-ASLD® program in Spanish children with autism and preterm children with communication and/or socialization difficulties | Valencia et al. (2021) |
| **Case studies**  **(N=7)** | Preventing Post Traumatic Stress Disorder in the general population induced by trauma during the COVID pandemic A simple brief intervention based on cognitive science that could be delivered digitally | Gargot et al. (2021) |
|  | COVID-19 phobia in a boy with undiagnosed autism spectrum disorder A case report | Sakamoto et al. (2021) |
|  | Reflections of COVID-19 Pandemic on Autism Spectrum Disorder: A Descriptive Case Study | Sani-Bozkurt et al. (2021) |
|  | Autism and COVID-19: A Case Series in a Neurodevelopmental Unit | Nollace et al. (2020) |
|  | How to train a child with Autism Spectrum Disorder to write persuasive texts. A case study during the lockdown caused by Covid-19 | Melogno et al. (2021) |
|  | Pregnancy outcomes among symptomatic and asymptomatic women infected with COVID-19 in the west of Iran: a case-control study | Jenabi et al. (2021) |
|  | Lessons learned: What the COVID-19 global pandemic has taught us about teaching, technology, and students with autism spectrum disorder | Roitsch et al. (2021) |
| **Reviews**  **(N=13)** | A systematic review of technological approaches for autism spectrum disorder assessment in children: Implications for the COVID-19 pandemic | Dahiya et al. (2021) |
|  | A systematic review of remote telehealth assessments for early signs of autism spectrum disorder: Video and mobile applications | Dahiya et al. (2020) |
|  | Epidemiology of Autism Spectrum Disorders: A Review of Worldwide Prevalence Estimates Since 2014 | Chiarotti & Venerosi (2020) |
|  | Telehealth and autism prior to and in the age of covid-19: A systematic and critical review of the last decade | Ellison et al. (2021) |
|  | Global prevalence of obesity, overweight and underweight in children, adolescents and adults with autism spectrum disorder, attention-deficit hyperactivity disorder: A systematic review and meta-analysis | Li et al. (2020) |
|  | Information and Communication Technologies to Support Early Screening of Autism Spectrum Disorder: A Systematic Review | Desideri et al. (2021) |
|  | Suicide, self-harm and thoughts of suicide or self-harm in infectious disease epidemics: a systematic review and meta-analysis | Rogers et al. (2021) |
|  | Effects of Diet, Nutrition, and Exercise in Children With Autism and Autism Spectrum Disorder: A Literature Review | Doreswamy et al. (2020) |
|  | Families With Children With Neurodevelopmental Disorders During COVID-19: A Scoping Review | Shorey et al. (2021) |
|  | Psychological eHealth interventions for people with intellectual disabilities: A scoping review | Oudshoorn et al. (2021) |
|  | Implementation of Telehealth Services to Assess, Monitor, and Treat Neurodevelopmental Disorders: Systematic Review | Valentine et al. (2021) |
|  | Psychological and Behavioral Impact of Lockdown and Quarantine Measures for COVID-19 Pandemic on Children, Adolescents and Caregivers: A Systematic Review and Meta-Analysis | Panda et al. (2021) |
|  | Social robots in hospitals: A systematic review | González-González et al. (2021) |
| **Duplicated that were not previously removed**  **(N=2)** | Protocol for a randomized pilot study (FIRST STEPS): implementation of the Incredible Years-ASLD® program in Spanish children with autism and preterm children with communication and/or socialization difficulties | Valencia et al. (2021) |
|  | Psychiatric disorders in children and adolescents during the COVID-19 pandemic | Palacio-Ortiz et al. (2020) |

S2: Basic data of the studies excluded in the eligibility phase

| **Reason** | **Title** | **Authors (year)** |
| --- | --- | --- |
| **No cross sectional/longitudinal studies**  **(N=10)** | Including children with developmental disabilities in the equation during this COVID‑19 pandemic | Aishworiya et al. (2021) |
|  | Editorial Perspective: COVID-19 pandemic-related psychopathology in children and adolescents with mental illness | Jefsen et al. (2021) |
|  | Supporting children with neurodevelopmental disorders during the COVID-19 pandemic | Summers et al. (2021) |
|  | The impact of the COVID-19 pandemic on children with autism spectrum disorders | Bellomo et al. (2020) |
|  | Promoting physical activity for children with autism spectrum disorders during coronavirus outbreak: Benefits, strategies, and examples | Yarımkaya & Esentürk (2020) |
|  | COVID-19: Overcoming the challenges faced by individuals with autism and their families | Eshraghi et al. (2020) |
|  | Covid-19 pandemic and impact on patients with autism spectrum disorder | Baweja et al. (2021) |
|  | Blurring boundaries: the invasion of home as a safe space for families and children with SEND during COVID-19 lockdown in England | Canning & Robinson (2021) |
|  | The impact of COVID‐19 on autism research: Reflections from China | Wang (2020) |
|  | A model of support for families of children with autism living in the covid-19 lockdown: Lessons from italy | Degli Espinosa et al. (2020) |
| **No intervention (N=3)** | Experiences of Parenting Child with ASD during COVID-19 Pandemic: A Cross-Sectional Study | Alyoubi & Alofi (2020) |
|  | Excessive and Problematic Internet Use During the Coronavirus Disease 2019 School Closure: Comparison Between Japanese Youth With and Without Autism Spectrum Disorder | Kawabe et al. (2020) |
|  | Psychological distress and burden among family caregivers of children with and without developmental disabilities six months into the COVID-19 pandemic | Iovino et al. (2021) |
| **Psychological, behavioral, and affective-emotional aspects are not analyzed**  **(N=11)** | Brief Report: Impact of COVID-19 on Individuals with ASD and Their Caregivers: A Perspective from the SPARK Cohort | White et al. (2021) |
|  | Conducting cbt for anxiety in children with autism spectrum disorder during covid-19 pandemic | Kalvin et al. (2021) |
|  | How filipino parents home educate their children with autism during covid-19 period | Cahapay (2020) |
|  | Parents’ strategies for home educating their children with autism spectrum disorder during the covid-19 period in zimbabwe | Majoko & Dudu (2020) |
|  | A parent-sibling dyadic interview to explore how an individual with Autism Spectrum Disorder can impact family dynamics | Critchley et al. (2021) |
|  | The Resilience of Social Service Providers and Families of Children With Autism or Development Delays During the COVID-19 Pandemic-A Community Case Study in Hong Kong | Wong et al. (2021) |
|  | Core experiences of parents of children with autism during the COVID-19 pandemic lockdown | Latzer et al. (2021) |
|  | Making a brochure about coronavirus disease (COVID‐19) for children with autism spectrum disorder and their family members | Kawabe et al. (2020) |
|  | While quarantined: An online parent education and training model for families of children with autism in China | McDevitt (2021) |
|  | Impact of containment and mitigation measures on children and youth with ASD during the COVID-19 pandemic: Report from the ELENA cohort | Berard et al. (2021) |
|  | Factors affecting the behavior of children with ASD during the first outbreak of the COVID-19 pandemic | Nuñez et al. (2021) |
| **Wrong population (N=6)** | The impact of COVID-19 on stress, anxiety, and coping in youth with and without autism and their parents | Corbett et al. (2021) |
|  | Perceptions of Families of Individuals with Autism Spectrum Disorder during the COVID-19 Crisis | Manning et al. (2021) |
|  | Priority concerns for people with intellectual and developmental disabilities during the COVID-19 pandemic | Tromans et al. (2020) |
|  | Brief report: The impact of the COVID-19 pandemic on health behaviors in adolescents with Autism Spectrum Disorder | Garcia et al. (2021) |
|  | Mental health of children with neurodevelopmental disorders during COVID-19: A brief report of family experiences from a low and middle income country | Kaku (2022) |
|  | Autistic traits and negative emotions in the general population during COVID-19: Mediating roles of the behavioural immune system and COVID-19 risk perception | Zhao et al. (2021) |
| **Duplicate or not found**  **(N=5)** | Autism Spectrum Disorder: Guidelines for management during the period of Social Isolation by Coronavirus (COVID-19) | Echavarria-Ramirez et al. (2020) |
|  | Editorial perspective: Covid‐19 pandemic‐related psychopathology in children and adolescents with mental illness | Jefsen et al. (2020) |
|  | Brief report: Impact of covid-19 on individuals with asd and their caregivers: A perspective from the spark cohort | White et al. (2021) |
|  | Perceptions of families of individuals with autism spectrum disorder during the covid-19 crisis | Manning et al. (2020) |
|  | Comparing the initial impact of COVID-19 on burden and psychological distress among family caregivers of children with and without developmental disabilities | Chafouleas & Iovino (2021) |
